# Widespread population decline in South America correlates with mid-Holocene climate change

**DOI:** 10.1038/s41598-019-43086-w

**Published:** 2019-05-09

**Authors:** Philip Riris, Manuel Arroyo-Kalin

**Affiliations:** UCL Institute of Archaeology, 31-34 Gordon Square, London, WCH1 0PY United Kingdom

**Keywords:** Palaeoecology, Population dynamics, Climate-change adaptation, Climate-change impacts, Tropical ecology

## Abstract

Quantifying the impacts of climate change on prehistoric demography is crucial for understanding the adaptive pathways taken by human populations. Archaeologists across South America have pointed to patterns of regional abandonment during the Middle Holocene (8200 to 4200 cal BP) as evidence of sensitivity to shifts in hydroclimate over this period. We develop a unified approach to investigate demography and climate in South America and aim to clarify the extent to which evidence of local anthropic responses can be generalised to large-scale trends. We achieve this by integrating archaeological radiocarbon data and palaeoclimatic time series to show that population decline occurred coeval with the transition to the initial mid-Holocene across South America. Through the analysis of radiocarbon dates with Monte Carlo methods, we find multiple, sustained phases of downturn associated to periods of high climatic variability. A likely driver of the duration and severity of demographic turnover is the frequency of exceptional climatic events, rather than the absolute magnitude of change. Unpredictable levels of tropical precipitation had sustained negative impacts on pre-Columbian populations lasting until at least 6000 cal BP, after which recovery is evident. Our results support the inference that a demographic regime shift in the second half of the Middle Holocene were coeval with cultural practices surrounding Neotropical plant management and early cultivation, possibly acting as buffers when the wild resource base was in flux.

## Introduction

The initial human colonisation of South America was a rapid process that led to the dispersal of hunter-gatherer populations to every major biome on the continent within a few millennia, starting at the latest around 14 k calendar years before present (cal BP). Colonising groups successfully adapted to a broad range of environments during the Terminal Pleistocene and early Holocene, from the Amazonian rainforest and Patagonian grasslands to the high Andes^1–4^. The genetic and demographic structure of early populations have been the focus of substantial recent research^5,6^. In parallel, a growing body of archaeological evidence from several regions has suggested that climatic transitions acted as a driver of significant regional depopulation during the mid-Holocene. Discontinuities in archaeological records have specifically been linked to increasingly unpredictable climatic regimes around this transition. Abandonment or retreat to refugia is suggested to have occurred in central Amazonia^7^, the south-central Cordillera^8,9^, eastern Brazil^10^, the Sabana de Bogotá^11^ and the Puna de Atacama^12^. The inferred existence of mid-Holocene demographic regime shifts in these widely distributed environments indicates that exogenous factors influenced human populations across the continent concurrently at this time.

**Figure 1:**
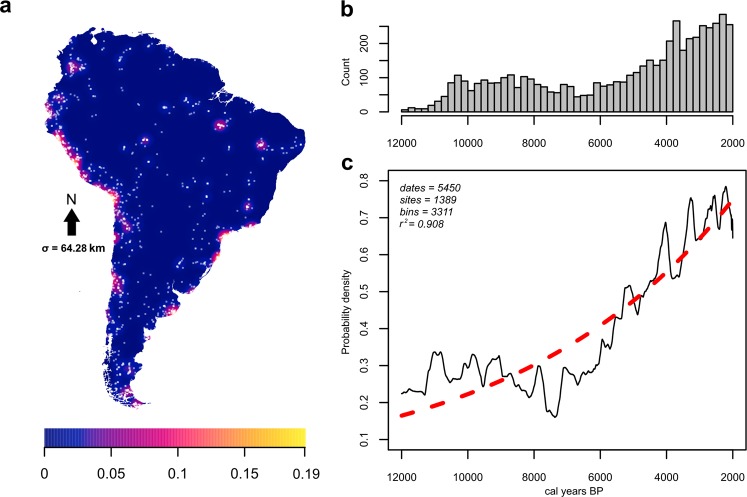
Archaeological sites and radiocarbon data: (**a**) Kernel-smoothed intensity of sites (white dots) for 12–2 k ^14^C years before present, measured in points/km^2^, (**b**) Histogram of median calibrated radiocarbon ages placed in 200-year bins, (**c**) Summed probability distribution of calibrated radiocarbon dates for entire South American dataset with a 100-year rolling mean (black solid line), shown with the highly correlated exploratory model fitted to data (exponential red dotted line), *R*^*2*^ = 0.971, Pearson.

Here we investigate pre-Columbian demographic dynamics to investigate the resilience of early South American foraging adaptations to periods of abrupt climate change. We focus specifically on the initial transition to the Middle Holocene (8.2–4.2 k cal BP^13^), during which South America was characterised by overall more arid conditions^14^. Large-scale analyses using of South American radiocarbon data as a population proxy have previously noted exceptionally low relative population around ~8.2 k cal BP^5^. We posit a connection between sudden, high-amplitude alterations to hydroclimate and widespread archaeological evidence of upheaval among human populations associated to the mid-Holocene transition. Globally, demographic overturn together with climate change has been suggested as a major driver of prehistoric culture change over this interval, with radiocarbon data proving especially instrumental in this regard^15–18^. In South America, the broad range of research intensities, historical trends in scholarship, preservation conditions, and site formation processes, against the backdrop of its cultural and ecological diversity^3^, requires any analysis to be undertaken in explicitly quantitative terms. We attend to this by assessing relative change in prehistoric South American demography using summed probability distributions of calibrated radiocarbon dates (Fig. 1, hereafter SPDs) combined with Monte Carlo simulation as an indirect proxy for demographic patterns over time^19–21^. To contextualise these findings, we also identify the frequency of hydroclimatic anomalies during the mid-Holocene across multiple palaeoclimatic time series (Methods), with the assumption that ecological shifts of direct consequence to human adaptations will track hydroclimatic regimes.

**Figure 2:**
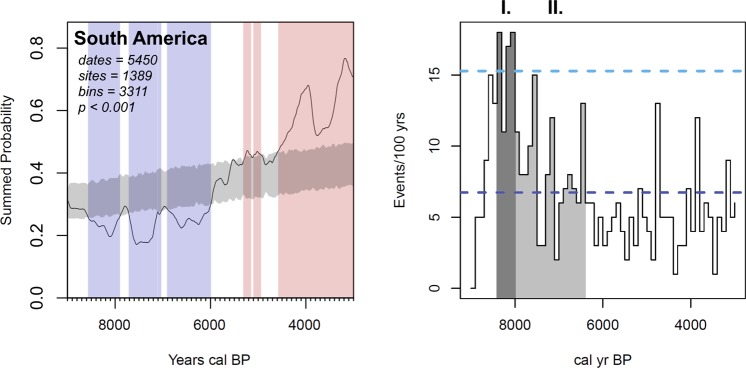
Test of summed probability distribution of calibrated archaeological ^14^C dates against a null model (grey shading) and climatic variability index. *Left*: Starting at 8.6 k cal BP South America experiences three phases of significant population deflation (blue shading). By the end of the mid-Holocene, the continental summed probability curve exceeds the null model (red shading). *Right*: (I) Exceptionally high climatic variability characterises the beginning of the mid-Holocene, with three time steps within 200 years of 8.2 k cal BP having an incidence of anomalies more than two standard deviations (light blue dashed line) above the mean (dark blue dashed line). (II) A second phase of cyclical high variability persists in the early mid-Holocene until 6.5 k cal BP.

We initially test the radiocarbon record of South America against the same null hypothesis of exponential growth for the period 12–2 k ^14^C years before present, based on an assessment of curve shape and goodness-of-fit. Following the detection of statistically significant negative departures from this model starting at ~8.2 k cal BP, we expand our approach through a two-phase demographic model with the goal of pinpointing where a demographic regime change is most likely to start (Fig. 2). Next, following preceding research^22^, we define our mid-Holocene demographic expectations with reference to prevailing dynamics prior to the phase of decline in order to condition our expectations of what constitutes a significant departure against the prevailing trend identified in the prior regime.

We also disaggregate the data into three regions for further analyses, comprised of: (a) the northern and central Andean Highlands, foothills, and foreland basin, (b) the tropical Lowlands of Amazonia and circum-Amazonia, and (c) the Southern Cone, incorporating the southern Andes, Pampas, and Patagonia. These subdivisions target variation in the structure of the archaeological data within broad topographic, biogeographical, and climatic realms. With reference to the systems driving rainfall variability across South America (Supplementary Information), these regions respectively approximate areas principally influenced by moisture transportation from the Atlantic, areas influenced by the Atlantic and potentially Pacific sources, and predominantly South Pacific/South Atlantic-influenced zones^13,23–25^. The Southern Cone, as defined here, also acts as a geographical proxy for the southern limits of tropical domesticates prior to the beginning of the late Holocene^6,26^. We take consistent patterns in the radiocarbon data to reflect robust and independent trends in human population across regions rather than specific archaeological cultures. These patterns permit identification of demographic sensitivity to climatic variability on a scale below that of the continent as a whole (Methods). We discuss the behavioural and social consequences of climatic variability during the mid-Holocene for the pre-Columbian population of South America based on the available archaeological data, as well as the role of climate as a driver of cultural change in general.

## Results

Our results show that the demographic trend for South America falls significantly below expectations for population growth after 8.6 k cal BP. After this point in time, periodic and statistically significant population deflation in the archaeological ^14^C record is apparent on a continental level, lasting at least until 6 k cal BP (p < 0.001, Fig. 2, left). This indicates that exceptional deviations from early Holocene demographic regimes occurred. The millennial-scale downturns associated to the initial Middle Holocene can be subdivided into three phases beginning with the initial dip in the demographic proxy at ~8.6 k cal BP, followed by ~7.7 k cal BP and finally ~6.9 k cal BP. These are bracketed by brief periods of recovery lasting two centuries or less. After the initial mid-Holocene, there is a return to model expectations, which are exceeded by ~5.3 k cal BP, likely marking the transition to a new demographic regime after ~6 k cal BP^5^. Our index of variability (Fig. 2, right) is derived from a robust outlier analysis of multiple palaeoclimatic records that together provide spatial coverage of precipitation patterns across South America. Our summary index shows a rapid increase starting at 8.6 k cal BP, concurrent with the first phase of downturn, peaking more than two standard deviations above the dataset mean at ~8.4 and 8.2–8.1 k cal BP. Following a short hiatus coincident with recovery, a second spike prefaces a second bout of demographic reduction after ~7.7 k cal BP. A third (~6.9–6 k cal BP) phase of sustained population decline spans the transition to more arid conditions that are typically recognised in the mid-Holocene of South America^23^. This is visible as a trough in our variability index punctuated by less intense yet above-average variability around 7.2 k and 6.5 k cal BP. Placing the frequency of hydroclimatic anomalies before and during the mid-Holocene transition alongside our demographic proxy illustrates the extent to which these patterns are coeval in time.

**Figure 3:**
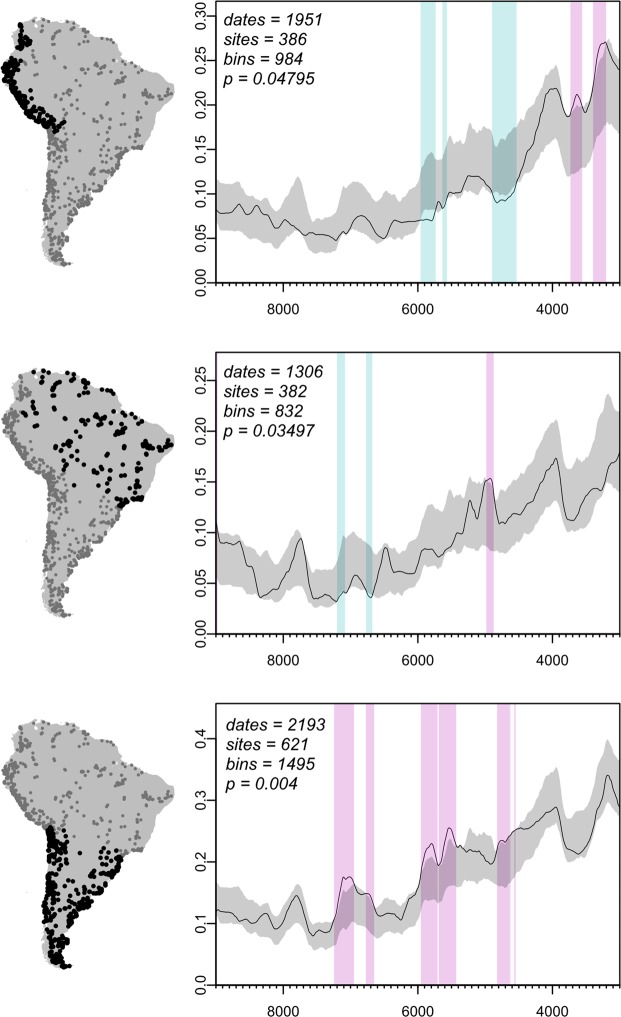
Permutation test of regional summed probability distributions, highlighting mid-Holocene asynchrony in the period 9 k – 3 k cal BP. Top: Tropical Highlands, the Northern Andes and Pacific Coast, Middle: Tropical Lowlands, Amazonia and circum-Amazonia, Bottom: Southern Cone, Southern Andes, Patagonia, and Pampas. Regional SPDs are compared against a 95% confidence envelope generated by randomly permuting the regional affiliation of each radiometric date (1000 runs). Significant deviations above (magenta) and below (cyan) the continental trend (95% confidence, grey) are asynchronous and frequently in antiphase between tropical South America and the Southern Cone.

The next set of analyses aims to identify spatial variability in demographic dynamics associated to the middle Holocene. Disaggregating the archaeological ^14^C record into three regions formally describes significant variation in the distribution and intensity of demographic downturns, underscoring that the impacts of climatic variability are themselves variable in space (Fig. 3). Negative and positive deviations in the permutation tests reflect periods where subsets of the data significantly exceed the overall continental trend^27^, indirectly confirming the existence of a continent-wide decline starting at ~8.6 k cal BP. It is important to note the reason for conformity between the summed probability distributions around 8.2 k cal BP. We find a lack of significant downturn in relation to the overall trend in the continental dataset consistent with the South America-wide downturn identified separately (see Fig. 2). As indicated by the null model test, the entire modelling domain is experiencing a demographic contraction at this time, which masks the statistical distinctiveness of coeval downturns in subsets of the data. The absence of a significant negative signal at this time in this test is therefore to be expected.

Together, the permutation tests reveal a staggered temporal structure in the summed probability distributions over the mid-Holocene chron. The tropical Highlands and Lowlands trends both show significant yet out of phase negative deviations from the continental confidence envelope. The Highlands appear responsive to heightened aridity around ~6 k and ~5 k cal BP, while declines in the Lowlands appear around 7 k cal BP. Inverse demographic trends exist between the Lowlands and Southern Cone at this time and between the Highlands and Southern Cone around 6 k cal BP. We observe that the most sustained local deviations in the Highland and Southern Cone data occur after 6 k cal BP, albeit opposite trends (negative and positive, respectively), while the Lowlands experiences only centennial-scale negative deviations. Pairwise regional comparisons with permutation tests (Fig. S2) concordantly show that the tropical Lowland and Highland SPDs return non-significant p-values, indicating statistical similarity, while both are significantly different from the Southern Cone. Together, our results indicate that a common mechanism may have influenced tropical South America, separately from the subtropical and temperate biomes of the Southern Cone. To this effect, we note significantly higher relative population in this region already by 7.5 k cal BP and repeatedly thereafter, with none of the negative phases experienced elsewhere.

In summary, we performed a parameter sweep on a broad range of possible null models for the Early and Middle Holocene. This indicated a critical breakpoint occurred at 8.6 k cal BP. To investigate the degree of departure from this stable (weakly linear) post-colonisation trend, we conditioned our null model on this data. When tested against simulated radiocarbon data generated with Monte Carlo methods, our results show a repeated and statistically significant downturn of varying duration and intensity after 8.6 k cal BP, concurrent with increasing climatic variability from this point in time. These phases last until at least 6 k cal BP, at which point a second regime shift appears likely^5^. Further testing reveals centennial-scale depressions in the archaeological ^14^C record are present across highly diverse environmental and cultural settings, revealing widespread population decline during the mid-Holocene. Regional differences are detected through comparison with the structure of the continent-wide dataset (Fig. 3), and identifies separate phases of downturn in the tropical Highlands and Lowlands data. In contrast, the Southern Cone remains largely above or within the continental confidence envelope. In statistical terms, the tropical Highlands and Lowlands reveal approximately equivalent demographic trends with some local differences, while the Southern Cone is significantly different from both. Significant positive divergences from the continental trend suggests that this region apparently did not suffer the same degree of downturn during periods of high climatic variability. Accounting for time-dependent site loss and spatial variability in preservation and research intensity (Methods), the weight of the statistical evidence suggests that the depth of the mid-Holocene downturn reflects much more than an oscillation of local population levels around a stable mean^5^. Rather, our results indicate that a phased demographic contraction over a period of several centuries took place across South America. Strong synchrony is evident between all three regions following peak climatic variability (Fig. 2). After the initial mid-Holocene, however, regional demographic responses diverge. Below we discuss possible impacts and consequences of this pattern in terms of the social and bioclimatic changes experienced by indigenous South Americans during this period of interest.

## Discussion

An initial period of high climatic variability spans the transition from the early to the middle Holocene. Three steps (8.4k, 8.2k, and 8.1 k cal BP) have an exceptionally high frequency of anomalies and the ramping up of frequent anomalous events shown in our index (Fig. 2) correlates with the initial drop in relative population observed across South America at and after 8.6 k cal BP. Archaeologists have repeatedly pointed to mid-Holocene aridity across South America as a mechanism driving occupational hiatuses in multiple localities, indicative of abandonment or logistic range reductions tethered to more predictable resources^7–12,27^. As illustrated by our archaeological summed probability distributions, significant departures from the quasi-stable Early Holocene regime occurred at least until 6 k cal BP, in the form of several protracted periods of population decline. A second, attenuated phase of anomalous climatic events following the initial extreme phase reveals the continuing impact of climatic variability on human populations. Our results indicate that precipitation variability, as well as absolute reductions in moisture, may have acted as joint drivers of demographic change in leading up to and in the first half of the Middle Holocene^28,29^. Importantly, the point identified as a probable demographic regime shift here is independent of the palaeoclimatic records included in our index.

**Figure 4:**
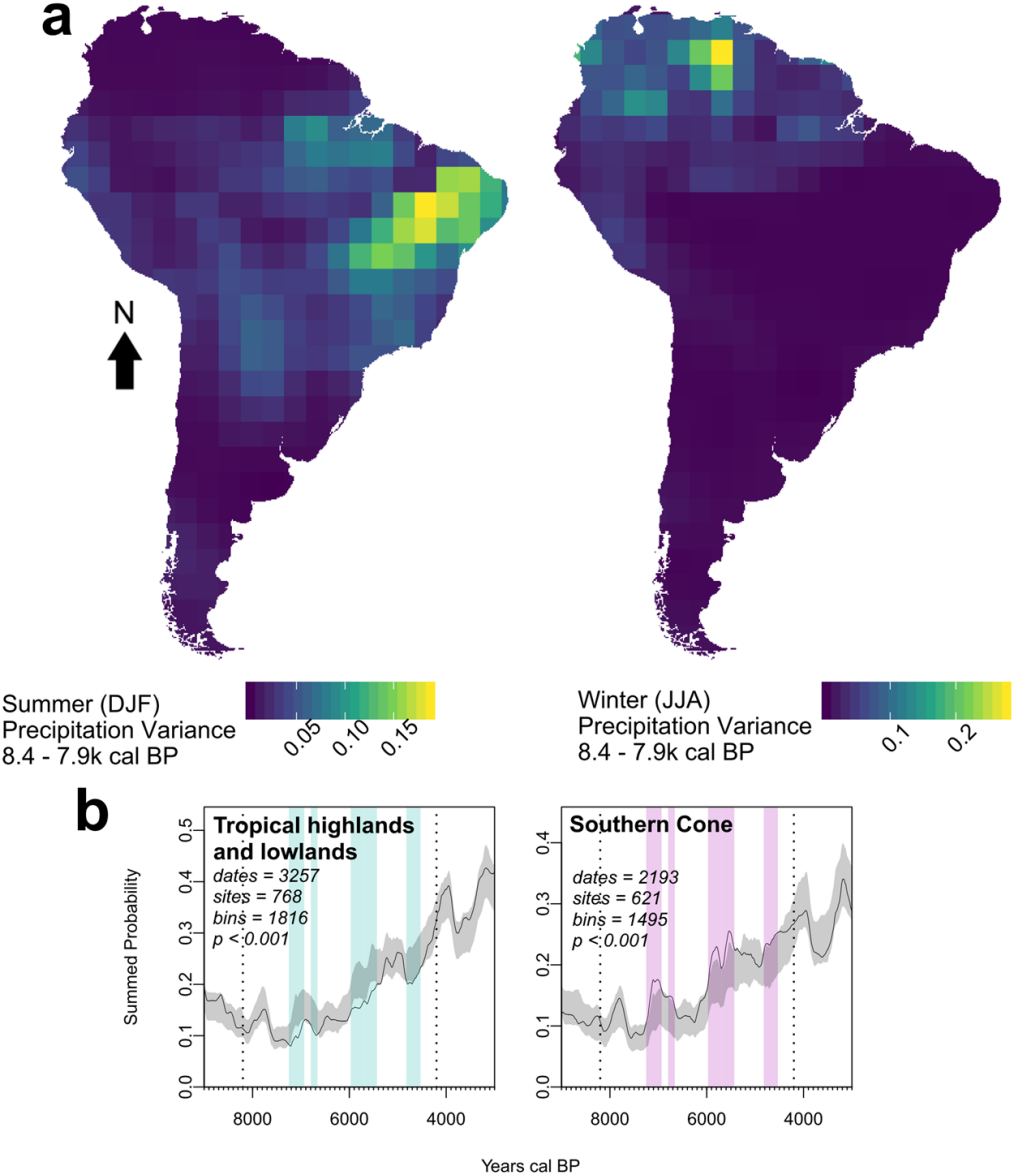
Correlating variance in Austral Summer (December-January-February) and Winter (June-July-August) precipitation during period of highest Mid-Holocene instability (8.4–7.9 k cal BP) and tropical versus extra-tropical demographic patterns. *Top*: Maps are based on 11 simulated grids of the TRaCE-21ka experiment in 50-year time steps in 50-year intervals. Grid cell resolution of the circulation model is 2.5°, projected to Albers Equal Area Conic for South America. The Southern Cone displays the overall lowest variance in precipitation over the mid-Holocene in both summer and winter. *Bottom*: Inverse demographic trends in the tropical highlands and lowlands during the Middle Holocene.

A key influence on summer monsoon precipitation is the seasonal procession of the Intertropical Convergence Zone (ITCZ) and its interaction with the South Atlantic Convergence Zone (SACZ) over tropical South America. As the magnitude of ITCZ movement southwards is ultimately driven by orbitally-forced changes in North Atlantic surface temperature, negative anomalies result in a northerly (<10 °S) mean latitude of the ITCZ^23^. This leads to a reduction in precipitation in eastern Brazil and southern Amazonia, generally in antiphase to wetter conditions in the northern and western portions of the continent during such events, including the tropical Andes^24,30^. Our selection of proxies (Fig. S3) provides a long-term average of variability in this mechanism in latitudinal cross-section across tropical South America, as well as a separate index of precipitation in the southern mid-latitudes of the continent, which are predominantly influenced by the relative strength of Pacific westerlies^31^. Furthermore, simulated precipitation grids (Fig. 4, top) suggest that the highest variance at the start of the mid-Holocene occurred in a broad arc across the tropics, from the north of the continent to eastern Brazil, inflected via western Amazonia. The central Amazon and circumscribed areas of the Pacific coast experienced the least variability in tropical precipitation patterns over this period. The latter agrees with the suppression of the ENSO phenomenon in the Pacific during the mid-Holocene^32^, although overall the southern latitudes of the continent present the least variable precipitation patterns. Although flora responded to overall more xeric mid-Holocene conditions, biome-scale vegetational transitions appear not to have been severe when averaged over approximately four millennia^30,33,34^. Foragers adapted to the diverse terminal- and post-glacial environments of South America^2,4^ consequently also reacted in varying ways.

We propose that a combination of factors was responsible for sustained demographic downturn evident in the summed probability distributions. Relatively sudden, high-amplitude variability in precipitation patterns acted as “climatic shocks”^35^ that provoked the downturns observable in our demographic proxy (Figs 2–4). Following initial spikes in climatic variability, depressed tropical moisture availability in the mid-Holocene^23,36^ may have caused small changes in precipitation to have disproportionate impacts on human-occupied niches. Populations that were adapted to early Holocene conditions may have first suffered negative impacts to their resilience, translating to increased vulnerability to comparatively small-scale climatic events after 8.2 k cal BP. Concordantly, where climatic instability is less pervasive, for example in the Southern Cone as defined here (Fig. 4, bottom), the negative effects on demography are lessened in comparison to regions where some degree of climatic oscillations endured, as in the tropical Highlands and Lowlands. Nonetheless, after 6 k cal BP, our archaeological ^14^C proxy in tropical regions is sufficiently recovered to be consistent with, or exceed, model expectations. The continental confidence envelope is surpassed in the Highlands by the onset of the early Holocene at 4.2 k cal BP, while the Lowlands do so well before this point in time (Fig. 3).

Amidst the geographically-variable effects of climate change, South American populations themselves enacted modifications to local ecosystems through the deployment of plant management and cultivation practices^37–40^. The precocious use and dispersal of comestible and useful plants began in the Early Holocene in South America, and the long-distance translocation of crops such as peanut (*Arachis hypogaea*), manioc (*Manihot esculenta*), and maize (*Zea mays*) by the mid-Holocene^40,41^ highlights the deep antiquity of cultivation practices in the Neotropics. These and other species were integrated into diversified and likely regionally-specific multi-crop procurement systems at various times after 8.2 k cal BP^37,39,42^. The deliberate and incidental use of fire to modify local ecologies also influenced and shaped the environmental niches of which these crops were a part^43–46^. We suggest that the development of these intertropical systems is a crucial development in the context of significant population reduction relative to earlier periods. Under the stresses induced by both demographic and environmental conditions, anthropic ecologies that already incorporated cultivated or managed resources before the mid-Holocene may have become proportionately more important to subsistence strategies in the face of an unstable climate^28,47^. The increasing visibility of cultivated plants in the palaeobotanical record from the mid-Holocene could suggest that climate change may have promoted the incorporation of a greater proportion of managed plants into tropical forager subsistence systems^40^. Population recovery during the second half of the mid-Holocene (after 6 k cal BP) is consistent with a florescence linked to a diversified and more stable resource base following climatic stabilisation to a drier yet steady phase^7,48–53^. A possible outcome of these cultural and environmental trajectories was the emergence, in some regions, of population aggregation and social institutions for the coordination and control of previously-unprecedented population densities among South American populations by the Late Holocene^26,47,50–55^.

Understanding mid-Holocene demographic patterns is predicated on our consideration of climatic variability, as well as against the backdrop of developments in cultivation practices. We follow recent research in noting that the scale of anthropic environmental legacies is necessarily linked to relative population over time^43,45,56^. Our examination of the archaeological and climatic record of South America provides a continental-scale framework for understanding the interplay between population dynamics and food procurement diversification over the mid-Holocene, as well as questioning at what point climate change or instability demands alternative pathways be adopted by human populations. In this regard, the broadening of the trophic niche of humans through the adoption of a greater proportion of plant resources may have functioned as a buffer against environmental unpredictability^6,28,29,40^. We can generalise that human populations in this period experienced significant and sustained periods of demographic downturn on a continental scale. These demographic processes were not uniform in either time or space and likely encompass substantial variation below the spatial scale we have adopted here. In particular, previously-identified local responses to mid-Holocene climate change require further investigation in the context of our findings.

The demographic signals highlighted on a broad scale in this work are composites of local archaeological records. Statistically significant deviations, whether negative or positive, invite further investigation into the trajectories adopted by human populations at a variety of spatial scales and settings. We anticipate that more research, ideally combining computational vegetation reconstructions and landscape modelling, will help to formally characterise how the demography of South America was shaped by, and in turn shaped, environmental conditions in the long term. Systematic assessments of cultural and biotic resilience to hydroclimatic variability are necessary to understand the development of both domains in the millennia following the human colonisation of South America.

## Methods

### Archaeological radiocarbon analysis

Our analysis employs a database of archaeological radiocarbon determinations compiled from a continuous and ongoing survey of the published academic and grey literature, with an especial focus on Amazonia. Our own collection is cross-referenced to large pre-existing databases^5,57^ and corrected with reference to the original published sources. Our data collection resulted in a set of 5450 radiocarbon determinations for the interval 12 k – 2 k ^14^C years before present. In contrast to previous compilations over this period^5^, we furnish a much larger sample of radiocarbon dates for Amazonia and circum-Amazonia, providing better control over this area.

We make use of the R package ‘rcarbon 1.2’^58^ to perform statistical analyses on our data. Following established frameworks for the aggregate analysis of archaeological ^14^C, we examine summed probability distributions (SPDs) of calibrated radiocarbon dates as a proxy for relative change in population over time. This approach to archaeological radiocarbon data rests on the assumption that higher past populations deposit more archaeological material to date, in turn resulting in the production of radiocarbon determinations commensurate with ancient demography^21,59–61^, i.e. an assumption of monotony between dated archaeological charcoal and past population levels. Sampling bias, time-dependent and spatially variable taphonomic loss, laboratory errors, calibration curve fluctuations, and sample contamination can potentially introduce systematic errors that obscure or exacerbate genuine demographic signals in the ^14^C record. Our mitigation measures for these issues are described in detail in the Supplementary Information. Deletion and loss of archaeological sites is unlikely to have operated on spatiotemporal scale sufficient to bias the record of an entire continent consistently^7,8,10,50^, and we take our ^14^C data to be broadly representative despite expectations of some localised site loss.

We initially perform the analysis on the entire South America dataset. However, the global trend may mask significant regional variation in potential subsets of the data. With reference to spatial structure, sites tend to be highly clustered in, for example, the desert coast of Peru, while being diffuse in the central Amazon basin, rendering a single clustering metric inappropriate for discovering viable subdivisions. Formal methods for grouping spatial point patterns such as k-means or density-based clustering are, respectively, unable to adapt to the arbitrary shape of the point pattern and the high variation in the spatial density of sites. For the purposes of the analysis, we choose to partition the South American radiocarbon data into three to investigate human population patterns and capture variation in the structure of the data within broad biogeographical and climatic realms, rather than any specific archaeological cultures or phenomena. The appearance of consistent patterns in the radiocarbon data should therefore reflect robust and independent cross-regional demographic trends. An objective of our study is to consider coeval shifts in demographic and climatic regimes at and around the transition to the Middle Holocene (8.2 k cal BP).

We have opted to divide all sites located above the 300 m elevation contour into northern and southern subsets along the Peru-Chile border, to form the core of the Highlands and Southern Cone datasets. Both sets include Pacific coast sites located to the west of the elevation cut-off point. We assigned dates from Bolivia in La Paz, Oruro, and Cochabamba departments to the Highlands, and those located in Chuquisaca, Tarija, and Potosí to the Southern Cone. The remainder of the Southern Cone consists of dates from Uruguay, Argentina, Paraguay, and the Brazilian states of Paraná, Rio Grande do Sul, and Santa Catarina. Sites located below 300 m above sea level and outside of the abovementioned elevation boundaries form the Lowlands dataset. These criteria produce three subsets of the data that group lowland Amazonia with the Guianas, the Orinoco basin, and northeast Brazil (here the Tropical Lowlands), the northern Pacific coast and Andes with the Amazonian foreland basin and foothills (here the Tropical Highlands), and finally the southern Pacific coast and Andes with the Pampas, Patagonia and the southern Brazilian highlands (the Southern Cone). With reference to the climatic systems that drive rainfall variability across South America (Supplementary Information), our subdivisions correspond approximately (in order) to areas principally influenced by moisture transportation from the Atlantic, areas influenced by the Atlantic and potentially Pacific sources, and predominantly South Pacific/Atlantic-influenced zones^13,23–25^. The Southern Cone also functions as a geographical proxy for the southernmost range of tropical domesticates before the late Holocene^6,26^.

The statistical modelling presented here principally concerns the mid-Holocene, but we carry out our analyses on dates in the interval 12–2 k cal BP. Dates pre-dating 12 k ^14^C years BP were excluded, as were those with Gaussian errors of >200 years. Although the initial peopling of South America was underway by 14 k cal BP, and possibly earlier^4^, the sparse evidence available for this period and its distance from the mid-Holocene makes it less germane to this study. Our initial analyses on the 5450 radiocarbon determinations acquired for the entire continent employ the following protocol:

i. *Calibration*. Radiocarbon dates are calibrated and aggregated by site into non-overlapping phases over the period 12–2 k ^14^C BP, and we report on the results from a focused time range of 9 k – 3 k cal BP. Aggregation of dates from the same site into bins of 200 years is carried out to account for the overrepresentation of well-dated sites. We principally make use of the SHCal13 curve^62^ for calibration (Fig. S4), except for determinations on marine shells, for which the Marine13 curve^63^ is used. This calibration curve is offset from the terrestrial curves by several centuries to account for the incorporation of ancient carbon from ocean upwelling into mollusc exoskeletons. In addition, we calibrate marine dates using local averaged ΔR offsets and errors by interpolating to the geographically-closest sampling site, acquired through an online repository^64^.
ii. *Summation*. The probability distributions of the calibrated dates are summed for the entire South American continent. We do not normalise the post-calibration probability distributions, to reduce the effect of peaks and plateaus in the calibration curves on the shape of the final SPDs^19^ (Sensitivity Analysis).
iii. *Model testing*. We initially test the South America-wide SPD against a simple fitted exponential population growth trend. Our choice is guided by an information criterion indicating maximum goodness-of-fit with this model over linear and logistic growth models (Sensitivity Analysis). A sample of calendar dates equal to the number of bins are drawn from the fitted model, converted to ^14^C dates, re-calibrated, and their probability distributions summed. We opt to draw from the uncalibrated date distribution. Errors for the re-calibration were generated by sampling with replacement from the empirical ^14^C errors of both marine and terrestrial dates^58^. Through a Monte Carlo procedure of 1000 runs, we derive confidence intervals for the null model.
iv. *Regional permutation tests*. Through the random assignation of marks to each date in the complete dataset, in this case the regional affiliation, a distribution of simulated SPDs are generated from 1000 Monte Carlo runs^27^, from which 95% confidence intervals are derived. The empirical SPDs were directly compared with the pan-regional trend produced from permuting the marks of the full dataset (n = 5450). We also performed pairwise comparisons of each of the three regions (Fig. S2).

The above procedures controls for the global effects of taphonomy and first-order spatial processes such as sea level rise, as exponential model fitting mimics the effects of time-dependent loss and the permutation tests makes use of the ^14^C determinations directly, in effect integrating the effects of taphonomic loss into the analysis^27,65^. Our initial results with this exploratory null model suggest statistically significant relative population decline during the Middle Holocene. Nonetheless, the initial formulation of the null hypothesis may be responsible for this finding (a Type I error) and thus we extend our model testing further to resolve this issue.

We follow Silva and Vanderlinden^22^ in specifying a null model based on prevailing patterns *prior* to a target period of interest rather than the dataset as a whole. Through inspection of our exponential model (Fig. 1), we first note that this null hypothesis performs particularly poorly in the Terminal Pleistocene and Early Holocene, and second, the SPD suggests a slow decline in the South American ^14^C record may begin already around 9 k cal BP. Further testing (Supplementary Information) indicates that the point of divergence from approximately stable, weakly linear Early Holocene demographic regimes^5^ is most likely to have occurred at 8.6 k cal BP (Fig. S1), providing a point of departure for investigating the degree to which mid-Holocene demographic trends diverge from prior patterns. That is, instead of a single null hypothesis for 12–2 k cal BP, we condition our new null model on the period 12–8.6 k cal BP to satisfactorily identify the degree of deviation after this point in time.

Our protocols allow us to examine salient features of the South American radiocarbon record, specifically: (a) the degree and significance of deviation in demographic trends from the null hypothesis of steadily increasing population throughout the Early Holocene in our domain, and (b) the similarity of population trajectories in each regional setting. Both sets of tests permit local and global tests of significance to be estimated, and regional population histories to be compared through z-transformation of the empirical and simulated SPDs^58^. We plot the null models against the empirical SPDs with a running average over a century applied to smooth out artefacts of the calibration process in the probability distributions.

### Climate variability index

To our knowledge there is no single prevailing criterion for defining a climatic anomaly, with a variety of thresholds, methods, and selection procedures reported in the literature^66^. To derive a robust index of climate variability we use the Median Absolute Deviation (MAD), a measure of statistical dispersion, on a set of precipitation records with near-complete latitudinal coverage of South America (Fig. S3). MAD provides a symmetrical estimate of the central tendencies of a time series, and effectively accounts for the value of any given data point in the context of a rolling window. It is not sensitive to isolated extreme outliers or non-normal distributions, and therefore correctly identifies sudden, large-amplitude changes and oscillations while excluding general trends. Here, we impose ±3 times the rolling MAD as a conservative threshold for identifying an extreme outlier. We scale the window to the resolution of each individual palaeoclimatic record to approximate a 100-year rolling average and have selected records with a resolution sufficient to allow a minimum of three points per interval. Where records have multiple sources of data, for example two speleothems reported from Lapa Grande cave, Brazil, the outlier analysis has been performed separately on these sources and subsequently combined.

We sum the identified anomalies from each record into 100-year bins to present them on a common time scale of calendar years before present (cal BP) and produce an intuitive summary index of climatic outliers for continental South America. In addition to the high initial MAD threshold for defining an abrupt event in each individual record, we suggest that frequencies of binned anomalies that are two standard deviations over the dataset mean are indicative of significantly above-normal climatic variability. Finally, we underline that the results simply reflect the sum of anomalies in a given time step, not their magnitude, spatial distribution, or whether they are negative or positive deviations from the rolling MAD of a given precipitation record. Deriving a composite measure of climatic variability aims to contextualise our demographic data (Figs 2 and 3) rather than directly infer the state of the climate or environment itself. We rely on the interpretations palaeoclimatologists and palaeoecologists to understand the conditions and impacts of climate on humans in our modelling domain.

## Supplementary information

Supplementary Information for Widespread population decline in South America correlates with mid-Holocene climate change

Dataset 1
