# Supplementary material for: Widespread population decline in South America correlates with mid-Holocene climate change
Source: Sci Rep. 2019 May 9;9:6850. doi: 10.1038/s41598-019-43086-w (PMC6509208; doi:10.1038/s41598-019-43086-w)
Supplement: Supplementary file 1 — Supplementary Information for Widespread population decline in South America correlates with mid-Holocene climate change [file 41598_2019_43086_MOESM1_ESM.pdf]

# Supplementary Information for
# Widespread population decline in South America correlates with mid-Holocene climate change

Philip Riris and Manuel Arroyo-Kalin

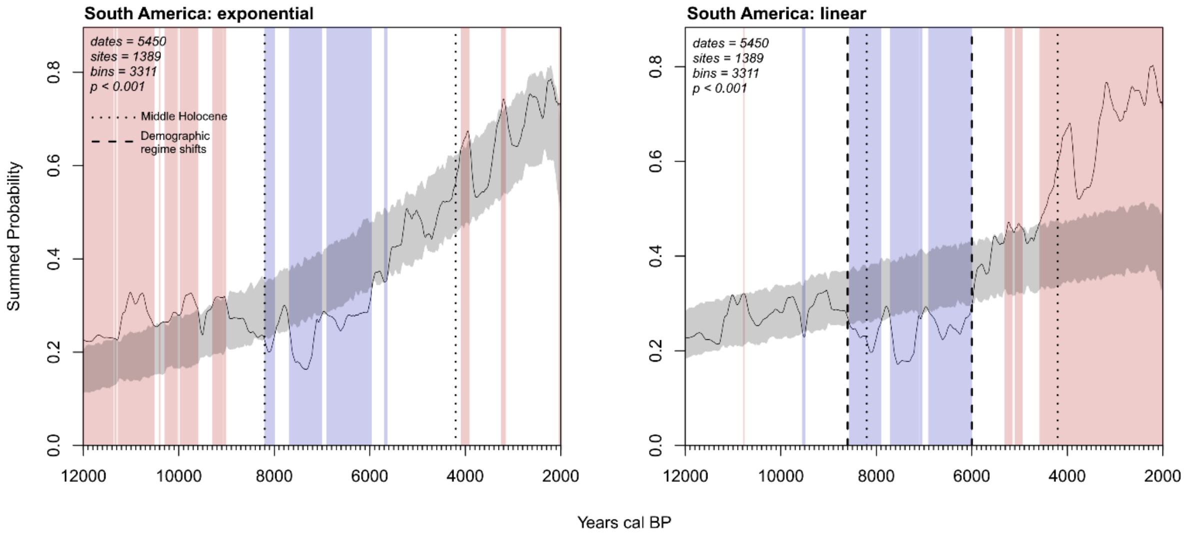


**Figure S1: Null hypothesis testing of the summed probability distributions of calibrated South American radiocarbon dates.** *Left:* A null hypothesis of exponential growth for the period 12 – 2k calendar years before present identifies significant downturn at between ~8.2 – 6k cal BP, but underestimates Terminal Pleistocene and Early Holocene population. *Right:* An improved statistical fit for the Early Holocene identifies a probable demographic regime shift starting at 8.6k cal BP, providing a point of departure for identifying Middle Holocene downturn. This pattern is coeval with increasing climatic variability after 8.6k cal BP, peaking at 8.2 and 7.9k cal BP. Based on the comparison of both null models, a second regime change is suggested to begin after ~6k cal BP[1].

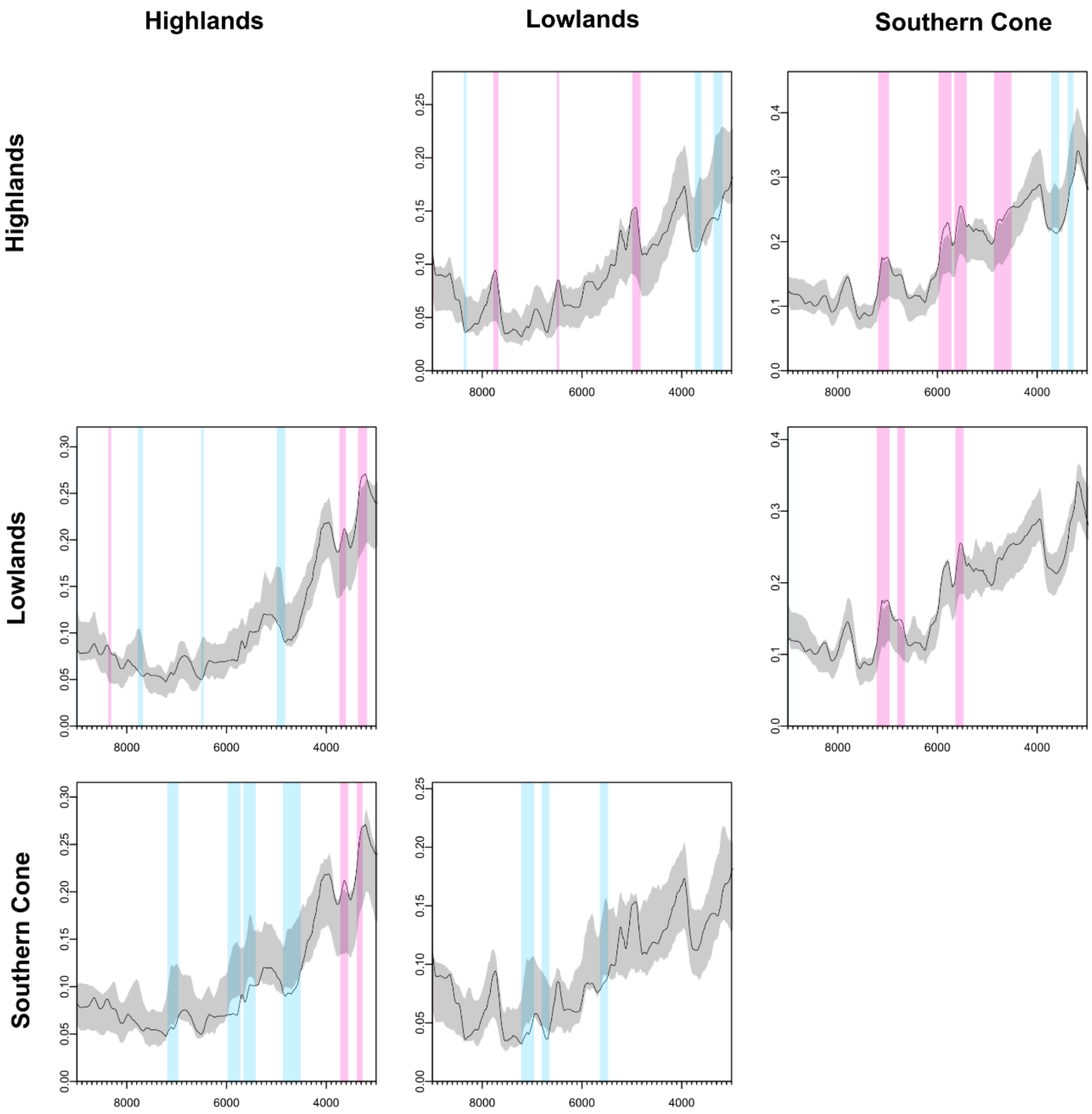


**Figure S2: Pairwise permutation tests of regional SPDs.** The Southern Cone subset shows the strongest differences from the Highlands and the Lowlands, with contrasting greater-than-expected deviations from both during the mid-Holocene. By the late Holocene, the Highlands supersede both the Southern Cone and the Lowlands, however, the global p-value indicates statistical equivalence between the tropical Lowlands and Highlands.

**Table S1: Regional pairwise permutation test of summed probability distributions.** P-values in *italics* are significant at the 0.05 level. Lowlands and Highlands are statistically equivalent, while the Southern Cone diverges significantly from both former regions globally; repeated periods of positive deviation can be observed in the Southern Cone during the mid-Holocene.

| | **Pairwise permutation test** | | |
|---|---|---|---|
| | **Highlands** | **Lowlands** | **Southern Cone** |
| **Highlands** | N/A | 0.1099 | *0.009* |
| **Lowlands** | 0.1189 | N/A | *0.003* |
| **Southern Cone** | *0.011* | *0.005* | N/A |

**Climate proxies and simulations**

Shifts in the regional climate of South America at the start of the mid-Holocene, for present purposes defined as 8.2 – 4.2k cal BP[2], are widely recognised in a range of independent archives. The interaction between the South Atlantic Convergence Zone (SACZ) and Intertropical Convergence Zone (ITCZ) determines the level and distribution of precipitation received by the majority of the continent, particularly through the South American Summer Monsoon (SASM). These mechanisms are likely driven by orbital forcing[3,4], which on millennial timescale has a latitudinal lag[5]. As the magnitude of ITCZ movement southwards is ultimately driven by orbitally-forced changes in North Atlantic surface temperature, negative anomalies result in a northerly (<10°S) mean latitude of the ITCZ[6]. This leads to a reduction in precipitation in eastern Brazil and southern Amazonia, a pattern which is in antiphase to wetter conditions in the northern and western portions of the continent during such events, including the tropical Andes[3,7]. Capturing hydroclimatic variability over time on the spatial scale adopted here therefore requires the use of multiple climate archives. Our selection of sedimentary and geochemical proxies provides a latitudinal cross-section of tropical South America. We cross-reference six independent archives through a robust outlier analysis and summary measure (**Methods**), with near-complete coverage of the Middle Holocene, to this end. As precipitation patterns in the southern mid-latitudes of the continent are predominantly influenced by the relative strength of Pacific westerlies, we provide an additional record for this region (**Figure S3**). Together, these records present evidence for the onset of several centuries of marked variability with an inception centred on the eighth millennium before present. We briefly summarise each record here.

Laminae in the Cariaco Basin, off the north coast of Venezuela, provide an equatorial index of variation in the latitude of the ITCZ, which controls the distribution of precipitation across tropical South America, including the Andes[4]. This archive of terragenic (darker, low precipitation) versus biogenic (lighter, high precipitation) input also tracks independently-resolved variations in North Atlantic sea surface temperature[8]. The Cueva del Tigre Perdido and Botuverá speleothems[7,9,10] record SASM strength and hence precipitation variation in Amazonia and South-eastern Brazil based on change (δ) in the ratio of stable isotope values of $^{18}O$. We also use sedimentary records from Laguna Yanacocha to track tropical Andean relative moisture[11]. The ratio of calcium to titanium tracks runoff and hence hydrological variability in the lake catchment, with higher values reflecting lower lake levels and increased aridity[12]. The Lapa Grande speleothem provides an almost uninterrupted record of precipitation in eastern

Brazil, whose regime is generally in antiphase to the western sectors of tropical South America covered by our other choice of records[13]. To capture precipitation variation in the southern mid-

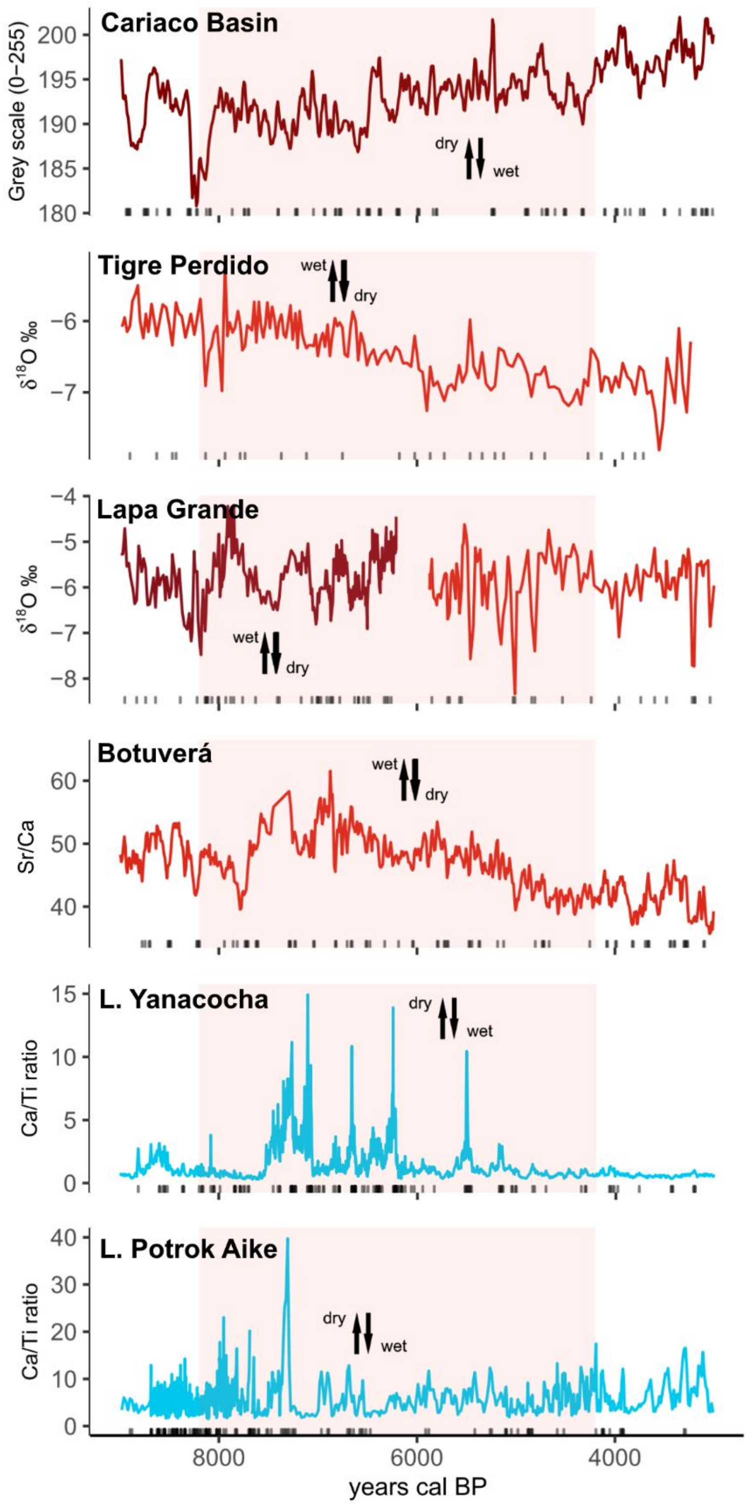


**Figure S3: Palaeoclimatic indices covering the period 9 -3k cal BP in South America. Top to bottom**: Cariaco Basin sedimentary reflectance data, high-resolution $\delta^{18}O$ values from speleothems in Cueva del Tigre Perdido and Lapa Grande, ratio of Strontium to Calcium from BTV21a at Botuverá, Lake Potrok Aike and Laguna Yanacocha sedimentary geochemistry.

latitudes, we finally consider sedimentary geochemical records from Lake Potrok Aike using the same chemical proxy as Laguna Yanacocha.

Separately, we also reconstruct the spatial distribution of inferred variability in mid-Holocene hydroclimate across South America using TRaCE-21ka data implemented in PaleoView 1.1. This data is derived from simulations run on the Community Climate System Model version 3 (CCSM3)[14,15], and couples flux from atmosphere-ocean-sea ice-land interaction to explain the evolution of global climate since the Last Glacial Maximum. The TRaCE-21ka experiment is capable of detecting both short-term features and long-term trends in regional palaeoclimate, and can successfully replicate modern conditions, for which there are accurate records to cross-reference against, to a high degree of correlation[16-18]. Output from TRaCE-21ka has previously been used to correlate archaeological phenomena with climatic events[19]. Within PalaeoView, user-defined spatio-temporal parameters are able to reconstruct past climatic conditions in time units as small as a month in decadal intervals[15], however, we choose to downscale to multi-decadal (50-year) intervals, and average over seasonal (austral summer and winter) precipitation patterns to match the phasing of the South American Summer Monsoon.

We model precipitation between 8.4 – 7.9k cal BP using the austral summer (December-January-August, DJF) and winter (June-July-August, JJA) as our time units in 2.5° grid cells between 15°N-60°S and 90-30°W. We set our benchmark relative to the early Holocene highstand of Lake Titicaca at 11.5k cal years BP, approximately coincident with the end of the Younger Dryas[4]. We first produce eleven 50-year time slices of mean daily precipitation (mm/day) over the study region for DJF and JJA with a step interval of 50 years (e.g. 4000-3951, 3950-3851 BP). We then standardise and rescale each grid to between -1 and 1, where negative values reflect less precipitation, positive the opposite, and zero is equivalent to conditions at 11.5k BP. Altering interval step or size parameters did not change the results markedly. This procedure produces a high-resolution spatial overview of South American hydroclimate for each step over the period of interest. Finally, assess individual grid cell variability, we calculate their variance for DJF and JJA between each set of eleven raster maps produced by the model. High values indicate large *differences* in precipitation over the period of interest, rather than arid or moist conditions in absolute terms. These simulations therefore directly complement the summary measure of variability we derived from the empirical palaeoclimatic time series outlined above (**Figure S3**).

**Sensitivity Analysis**

Our analyses employ several free or partially-free parameters: the kernel density visualisation requires a smoothing bandwidth (σ), while SPDs require a bin size (*h*), calibration employs one or more calibration curves, and the summed probability distributions a decision to sum to unity (normalise) or not. The following sensitivity analyses provide statistical justification for our selection of parameters, as well as the initial and subsequent null models against which we test our demographic proxy. Laboratory and reporting errors as potential sources of systematic bias in the $^{14}C$ database are mitigated by the size of our dataset, as the effects of any single source of error are smoothed by the quantity of dates lacking these sources of error. Absolute differences in the quantities of dates for each region are not an issue, as the output of all our radiocarbon analysis is z-transformed[20].

In our exploratory analysis (**Figure 1**) we fitted our data to exponential, linear, and logistic growth models for the period 12k – 2k cal BP, using Akaike's Information Criterion as a measure of model goodness-of-fit[21]. This selection criterion indicates the lowest value of ΔAIC for the exponential model among this set, with the next-best fit (logistic model) having a higher ΔAIC by a considerable margin at 4341.94. While this model is adequate for initially identifying the extent and significance of mid-Holocene demographic downturn, we are conscious that such a formulation of the null hypothesis may itself be implicitly responsible for the results. In the context of the European Neolithic, Silva and Vanderlinden[22] argue that establishing statistical expectations for demography over a period of interest must be made with reference to the prevailing conditions prior to that period, as opposed to a best fit for the span of the whole dataset. To accurately test for the presence and establishment of a different demographic regime, in this case downturns during the Middle Holocene (~8.2 – 6k cal BP per the exponential model), our null hypothesis ought to be the best fit for the period *prior* to the regime shift.

Unlike the European Neolithic, our modelling domain lacks an unambiguous archaeological marker of demographic regime change. Rather than arbitrarily selecting a cut-off point in time for our fit, we proceed with a stepwise assessment of a range of null models, employing AIC as a selection criterion as before. We fit a total of 122 two-phase piecewise models for the period 12 – 2k cal BP that permute through both linear and exponential fits with breakpoints in 50-year intervals over the period 10 – 7k cal BP, followed by an exponential fit[1] thereafter. Out of the fitted models (61 each of linear and exponential in the Early Holocene), the best

goodness-of-fit is obtained with a breakpoint of 8.6k cal BP, selecting a linear model whose slope is weakly positive in the interval 12 – 8.6k cal BP. In contrast to our initial exponential-only model, this model provides an excellent fit for the period of relative stability following initial human colonisation of South America (**Figure S1, right**), and identifies sustained negative deviations after approximately ~8.6k cal BP.

Recovery from population collapses in the second half of the Middle Holocene (~6k cal BP onward) was rapid and likely mediated by the increasing adoption of cultivated plants (see paper discussion). Goldberg et al.[1] suggest a regime shift to exponential growth in the second half of the Middle Holocene (**Figure S1, left**), we do not target this subsequent period of explosive growth implied by the summed probability distribution. We do not find statistical support for constant (uniform) relative population between 12 – 7k cal BP, which we attribute to using the non-normalised probability distribution[23], thus minimising extreme spikes and troughs (**Figure S4c**), as well as employing a more geographically-representative sample of dates and altering the curve as a result (**Figure 1**). We note that in this prior evaluation of prehistoric South American demography, the empirical probability distribution falls outside the 98% confidence interval at the mid-Holocene transition. Bearing this in mind, we emphasis that our model selection procedure, above, as well as all three of our initial exploratory null models (linear, exponential, logistic) identified significant negative divergence around 8.2k cal BP, validating our choice of model conditioned on Early Holocene data to examine the transition to the Middle Holocene. The second null hypothesis for South America bolsters the findings indicated by our exploratory efforts model and permits examination of the link between Middle Holocene hydroclimatic variability and demographic dynamics on a sound statistical basis.

We use likelihood cross-validation to select a sigma (σ) value for the kernel density estimate[23] (**Figure S4a**). Our sample consists of 1389 archaeological sites with unique spatial coordinates for the entire period of interest. Prior to analysis, we project the archaeological point pattern and its window, a base map of South America, to the continental Albers Equal Area Conic projection, and rescale our data to report the resulting kernel density estimate in points per km$^2$. The point process likelihood cross-validation criterion function:

$$\mathrm{LCV}(\sigma) = \sum_i \log(\hat{\lambda}_{-i}(x_i) - \int_W \hat{\lambda}(u)\mathrm{d}u$$

indicates a sigma value of 64.28 km is appropriate for the dataset. We use the default Epanechnikov smoothing kernel.

Rather than arbitrarily select a bin size, we permute through bin values from 0 to 500 in 50-year intervals to produce a visual index of variation in the resulting SPDs (**Figure S4b**). Overall, SPD shape remains similar except at extreme values of *h* (0 and 500 years). To compromise between the slight variation in shape and the median Gaussian error of ±70 years in the dataset, we elected to proceed with a bin size of 200 years for both the exponential model testing and permutation tests.

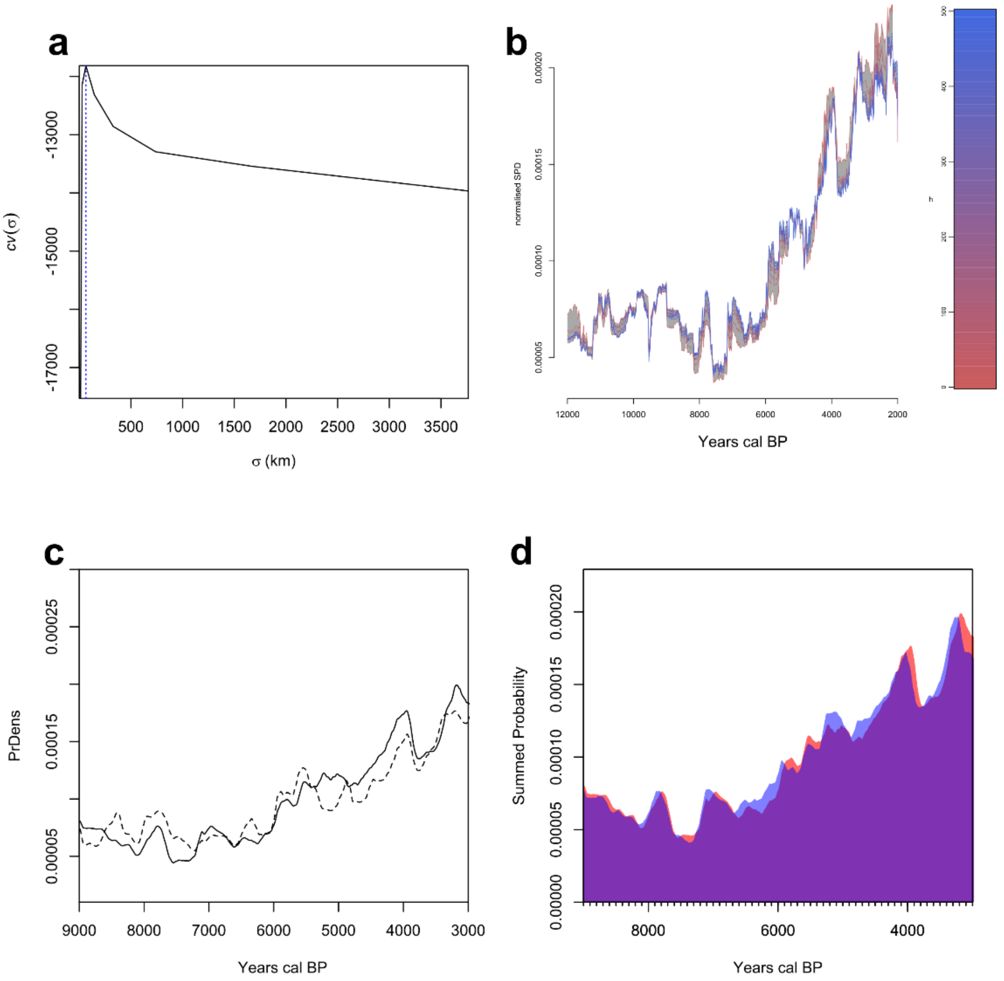


**Figure S4: Sensitivity analysis of parameter selection**: a) Likelihood cross-validation statistic for density plot, b) Effect of bin size on SPD shape, c) Normalised (dotted line) versus non-normalised SPD (solid line), d) Calibration curve effects on SPD shape using IntCal13 curve (blue) and without (red), with overlap (purple).

Post-calibration normalisation of radiocarbon dates has the effect of introducing artificial spikes and plateaus into the SPD along steep and flat segments of the calibration curve,

respectively[23,25], some of which can be substantial. **Figure S4c** illustrates this effect over the span of our data, showing several peaks and troughs where the non-normalised SPD has relatively gentle features, or even counter-indicates the normalised trend (e.g. near 8.5 and 5k cal BP). Given that our study aims to detect the existence and strength of significant deviations from model-derived expectations of demographic patterns in the mid-Holocene, normalisation could affect the robustness of the exponential model testing approach employed here. Following established precedent[23], we do not normalise the calibrated datasets or resulting SPDs.

Finally, a small proportion of our sample is located north of the equator (*n* = 344 dates, 5.92%). The seasonal movement of the Intertropical Convergence Zone (ITCZ) determines the relative proportion of northern versus southern atmospheric exchange, with commensurate variation in the uptake of $^{14}C$ in $CO_2$ by living organisms[26]. Choosing between calibration with the standard northern hemisphere curve[27] or the southern hemisphere curve[28] has a potential impact on subsequent analyses, as these curves are offset from each other by up to 20 years due to differing concentrations of atmospheric $^{14}C$. Furthermore, the seasonality and magnitude of latitudinal ITCZ migration has also varied considerably throughout the Holocene[8]. Seasonal and long-term orbital-driven movements of the ITCZ may therefore have introduced non-trivial variation in the quantity of $^{14}C$ in dated samples that is challenging to control for on a date-by-date basis. We produced an SPD employing both calibration curves, and another using only the southern hemisphere curve. These demonstrate little qualitative differences in their shape, showing the greatest differences after our period of interest, close to 3000 BP, possibly related to increased incidence of El Niño events in the later Holocene[5]. These two SPDs are highly correlated (**Figure S4d**, Pearson's $r = 0.993363$, $p < 0.001$). For all analyses we elect to employ the SHCal13 curve, except for determinations on marine molluscs. These are calibrated with the Marine13 curve, employing the regional offsets and errors outlined above[29].

## SI References


1. Goldberg, A., Mychajliw, A.M. & Hadly, E.A. Post-invasion demography of prehistoric humans in South America. *Nature* **532**, 232–235 (2016).
2. Walker, M.J.C. et al. Formal subdivision of the Holocene Series/Epoch: a Discussion Paper by a Working Group of INTIMATE. *J. Quat. Sci* **27**, 649-659 (2012).

3. Smith, R.J. & Mayle, F.E. Impact of mid-to late Holocene precipitation changes on vegetation across lowland tropical South America: a paleo-data synthesis. *Quaternary Research* **89,** 134-155 (2018).
4. Baker, P.A. et al. The history of South American tropical precipitation for the past 25,000 years. *Science* **291,** 640-643 (2001).
5. Schneider, T., Bischoff, T. & Haug, G.H. Migrations and dynamics of the intertropical convergence zone. *Nature* **513,** 45-53 (2014).
6. Deplazes, G. et al. Links between tropical rainfall and North Atlantic climate during the last glacial period. *Nature Geoscience* **6,** 213-217 (2013).
7. Cruz, F.W. et al. Orbitally driven east–west antiphasing of South American precipitation. *Nature Geoscience* **2,** 210-214 (2009).
8. Haug, G.H. et al. Southward migration of the intertropical convergence zone through the Holocene. *Science* **293,** 1304-1308 (2001).
9. Cheng, H. et al. Climate change patterns in Amazonia and biodiversity. *Nature Commun* **4,** 1411 (2013).
10. Bernal, J.P. et al. High-resolution Holocene South American monsoon history recorded by a speleothem from Botuverá Cave, Brazil. *Earth and Planetary Science Letters* **450,** 186-196 (2016).
11. Stansell, N.D. et al. Late Glacial and Holocene glacier fluctuations at Nevado Huaguruncho in the Eastern Cordillera of the Peruvian Andes. *Geology* **43,** 747-750 (2015).
12. Haberzettl, T. et al. Late glacial and Holocene wet–dry cycles in southern Patagonia: chronology, sedimentology and geochemistry of a lacustrine record from Laguna Potrok Aike, Argentina. *The Holocene* **17,** 297-310 (2007).
13. Stríkis, N.M. et al. Abrupt variations in South American monsoon rainfall during the Holocene based on a speleothem record from central-eastern Brazil. *Geology* **39,** 1075-1078 (2011).
14. Collins, W.D. et al. The community climate system model version 3 (CCSM3). *Journal of Climate* **19,** 2122-2143 (2006).
15. Fordham, D.A. et al. PaleoView: a tool for generating continuous climate projections spanning the last 21 000 years at regional and global scales. *Ecography* **40,** 1348-1358 (2017).
16. Otto-Bliesner, B. et al. Coherent changes of southeastern equatorial and northern African rainfall during the last deglaciation. *Science* **346,** 1223-1227 (2014).

17. Liu, Z. et al. Evolution and forcing mechanisms of El Niño over the past 21,000 years. *Nature* **515,** 550-553 (2014).
18. Harrison, S.P., Bartlein, P.J. & Prentice, I.C. What have we learnt from palaeoclimate simulations? *J. Quaternary Sci.* **31,** 363-385 (2016).
19. Gauther, N. The spatial pattern of climate change during the spread of farming into the Aegean. *J. Arch. Sci*. **75,** 1-9 (2016).
20. Crema, E.R. et al. Summed probability distribution of 14C dates suggests regional divergences in the population dynamics of the Jomon period in eastern Japan. *PLoS One* **11,** e0154809 (2016).
21. Sakamoto, Y., Ishiguro, M. & Kitagawa, G. *Akaike Information Criterion Statistics*. Tokyo: KTK Scientific Publishers (1986).
22. Silva, F. & Vanderlinden, M. Amplitude of travelling front as inferred from $^{14}C$ predicts levels of genetic admixture among European early farmers. *Scientific Reports* **7**, 11985 (2017).
23. Bevan, A. et al. Holocene fluctuations in human population demonstrate repeated links to food production and climate. *Proc. Natl Acad. Sci. USA* **114,** E10524-E10531 (2017).
24. Baddeley, A., Rubak, E. & Turner, R. *Spatial Point Patterns: Methodology and Applications with R*. London: Chapman and Hall/CRC Press (2015).
25. Weninger, B. et al. Quantum theory of radiocarbon calibration. *World Archaeology* **47,** 543-566 (2015).
26. Ogburn, D.E. Reconceiving the chronology of Inca imperial expansion. *Radiocarbon* **54,** 219-237 (2012).
27. Reimer, P.J. et al. IntCal13 and Marine13 radiocarbon age calibration curves 0–50,000 years cal BP. *Radiocarbon* **55,** 1869-1887 (2013).
28. Hogg, A.G. et al. SHCal13 Southern Hemisphere calibration, 0–50,000 years cal BP. *Radiocarbon* **55,** 1889-1903 (2013).
29. Reimer, R. *14CHRONO Marine Reservoir Database*. http://calib.org/marine/ (2018).
